# Phase-specific sequential press-needle therapy for menstrual migraine: a randomized trial protocol

**DOI:** 10.3389/fneur.2026.1876533

**Published:** 2026-09-11

**Authors:** Kun Hong, Xingyan Yang, Xiaomei Long, Xuemei Li, Lijuan Liu, Zhiqiang Zhang, Luhong Yu, Xue Fu, Zuoqin Yang, Xingping Xiao

**Affiliations:** 1 Department of Acupuncture, Chengdu Pidu District Hospital of Traditional Chinese Medicine, Chengdu, Sichuan, China; 2 Department of Rheumatology, Chongqing Hospital of Traditional Chinese Medicine, Chongqing, China; 3 Department of Pharmacy, Yongchuan People’s Hospital of Chongqing, Chongqing, China

**Keywords:** acupuncture, menstrual migraine, press-needle therapy, protocol, RCT

## Abstract

**Background:**

Menstrual migraine (MM) is a neurological disorder affecting women of childbearing age. According to the definition in the International Classification of Headache Disorders, 3rd Edition (ICHD-3), it includes pure menstrual migraine (PMM) and menstrually-related migraine (MRM). The efficacy and safety of acupressure therapy for menstrual migraine have not yet been fully evaluated, particularly when administered sequentially according to specific phases of the menstrual cycle. Therefore, we designed this trial to preliminarily assess whether sequential acupressure therapy shows a favorable trend in efficacy compared with oral naproxen sodium, and to evaluate its feasibility, safety, and tolerability, thereby providing baseline data for future large-scale confirmatory trials.

**Methods:**

This is a randomized, active-controlled, parallel-group trial. A total of 76 eligible women aged 18–45 years with migraine without aura and diagnosed with pure menstrual migraine or menstrually-related migraine will be recruited and randomly allocated to receive either sequential press-needle therapy or oral naproxen sodium therapy. In the press-needle group, treatment is delivered at designated acupoints across two phases (pre-menstrual and post-menstrual) per menstrual cycle. Each phase includes 2 sessions with a 2-day interval (4 sessions per cycle), repeated for 3 consecutive cycles. In the control group, oral naproxen sodium (550 mg twice daily after breakfast and dinner) is initiated 2–3 days before the expected onset of menstruation and continued until menstruation ends (5–7 consecutive days per cycle), also for 3 complete cycles. Compliance is monitored by a daily medication diary and telephone follow-up. The primary outcome is the change in the number of headache days with a peak pain intensity of ≥4 (moderate-to-severe) on a 0–10 Numeric Rating Scale (NRS, where 4–6 = moderate, 7–10 = severe) per menstrual cycle from baseline to the end of the 3-month intervention. Secondary outcomes include headache intensity, functional impact, quality of life, acute medication use, and heart rate variability indices. Exploratory outcomes include serum biomarkers and resting-state functional MRI.

**Conclusion:**

This trial will evaluate the preliminary efficacy and safety of sequential press-needle therapy compared with oral naproxen sodium for menstrual migraine. The results will be used to estimate effect sizes and inform the design of future adequately powered confirmatory trials.

**Study protocol registration:**

https://itmctr.ccebtcm.org.cn/mgt/project/view/2049072453099061248, ITMCTR2025002153.

## Introduction

Menstrual migraine (MM) is a neurological disorder with a high prevalence that significantly impacts women’s lives; it is characterized by migraine attacks concentrated around the menstrual period. According to the International Classification of Headache Disorders, 3rd Edition (ICHD-3), MM can be subdivided into two distinct types: pure menstrual migraine (PMM, Appendix code A1.1.1) and menstrually-related migraine (MRM, Appendix code A1.1.2). PMM is defined as migraine attacks occurring exclusively between the second day before menstruation and the third day after menstruation in at least two of three consecutive menstrual cycles, with no attacks at other times during the menstrual cycle. MRM refers to attacks that occur both within this perimenstrual window and at other times of the cycle in at least two of three cycles (1). These two subtypes reflect fundamentally different temporal patterns of estrogen-withdrawal susceptibility and trigeminovascular sensitization. Epidemiological studies indicate that up to 60% of female migraineurs experience menstrual-related attacks, which are often more severe, longer in duration, and less responsive to conventional acute medications compared to non-menstrual migraines (2). These headaches substantially impair daily functioning, reduce quality of life, and contribute to absenteeism and socioeconomic burden (3).

The pathophysiological mechanisms of MM involve multiple factors and are primarily driven by a sudden drop in estrogen levels. This process modulates serotonergic and glutamatergic neurotransmission, triggers the release of calcitonin gene-related peptide (CGRP), and exacerbates cortical hyperexcitability and central sensitization (4, 5). Given the predictability of attack timing, short-term perimenstrual prophylaxis (SPP) is the recommended management strategy. International guidelines, including those from the European Headache Federation, identify naproxen sodium as the first-line drug choice for SPP, as it has recognized efficacy in reducing the frequency and severity of menstrual migraines through potent inhibition of peripheral and central cyclooxygenase-2 (6). However, long-term use of naproxen and other nonsteroidal anti-inflammatory drugs (NSAIDs) is associated with gastrointestinal intolerance, an increased risk of bleeding, and the potential to induce medication-overuse headaches (7, 8). This highlights an unmet medical need for the development of safe, well-tolerated, and effective preventive treatment options.

Acupuncture has been increasingly applied for migraine prophylaxis, with evidence supporting its effect in reducing headache frequency and intensity, possibly through modulation of pain-related brain networks, autonomic balance, and neuroinflammatory markers (9). However, most existing acupuncture protocols are fixed-point regimens that do not account for the cyclic nature of menstrual headaches. Sequential acupuncture tailored to the menstrual phase—pre-menstrual and inter-menstrual—may better align with the dynamic hormonal and neurovascular changes, potentially enhancing therapeutic efficacy (10, 11). Furthermore, a significant practical barrier to conventional acupuncture is the need for frequent clinic visits, which can be time-consuming, burdensome, and lead to poor long-term adherence. Press-needle therapy, characterized by its semi-permanent indwelling needles that patients can self-stimulate, drastically reduces the frequency of clinic visits while providing continuous therapeutic stimulation (12). This feature may address key limitations of traditional acupuncture by improving treatment convenience, patient compliance, and overall acceptability—a crucial factor for the successful management of a chronic, cyclical condition like menstrual headache. Nevertheless, high-quality randomized trials evaluating such time-sensitive acupuncture approaches are scarce.

Given the limitations of current treatments and the theoretical rationale for phase-specific intervention, we designed a randomized trial to evaluate the efficacy and autonomic effects of sequential press-needle therapy for menstrual migraine. This protocol integrates objective biomarkers, heart-rate variability, and patient-reported outcomes to comprehensively assess treatment response and explore potential mechanisms.

## Objectives

The efficacy and safety of acupressure therapy for menstrual migraines have not yet been fully evaluated, and most existing acupuncture trials for MM have employed fixed protocols without phase-specific adjustments. Furthermore, no study has systematically integrated objective autonomic nervous system function, serum biomarkers, and neuroimaging findings to comprehensively assess the mechanisms underlying acupressure treatment for MM. To address these limitations, we designed this randomized exploratory trial to:

(1) Evaluate the feasibility of a phase-specific sequential acupuncture protocol;
(2) Assess the preliminary efficacy and variability of pressure-point acupuncture compared with the standard naproxen prophylaxis regimen, providing an effect size estimate for future sample size calculations;
(3) Explore potential mechanisms of action through multidimensional autonomic, serological, and neuroimaging markers.

Importantly, this trial is an exploratory rather than a confirmatory study. The findings will inform the design of future large-scale, definitive randomized controlled trials. We hypothesize that, compared with naproxen, sequential pressure-needle therapy will show a clinically favorable trend and demonstrate good safety and tolerability, and that the observed changes in autonomic nervous system and biomarker results will provide mechanistic insights for subsequent studies.

## Methods

### Trial design

**Figure 1:**
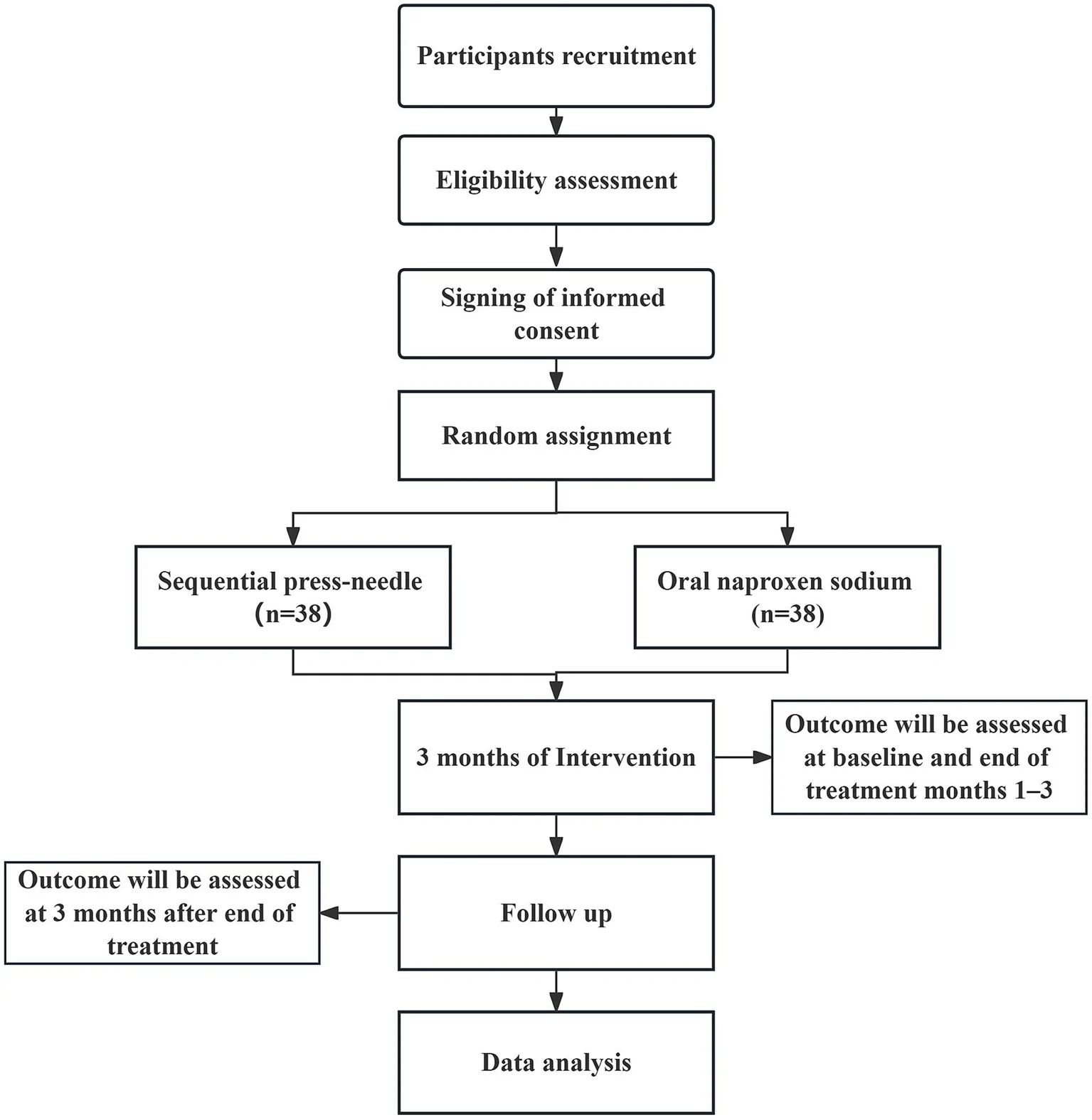
Flow chart for clinical trial procedure.

This research protocol was designed in accordance with the requirements of the 2025 version of SPIRIT guidelines (13). The flowchart of the entire trial process is provided in Figure 1. This figure details the paths of participant recruitment, screening, randomization, intervention measures, assessment, and analysis, and visually compares the intervention plans and key time points of the two study groups.

### Study participants

This single-center trial will be conducted at Chengdu Pidu District Hospital of Traditional Chinese Medicine. Participants will be diagnosed according to the ICHD-3 criteria. Eligibility assessment will be performed by board-certified neurologists or headache specialists. Written informed consent, obtained prior to any study procedures, is mandatory for enrollment.

### Eligibility criteria

#### Inclusion criteria

1. Meets the diagnostic criteria for “migraine without aura” (ICHD-3 code 1.1)” as defined in the International Classification of Headache Disorders, 3rd Edition (ICHD-3);
2. Meets the diagnostic criteria for “pure menstrual migraine” (A1.1.1) or “menstrually-related migraine” (A1.1.2) as outlined in the appendix to the ICHD-3, and this diagnosis has been validated by a prospective headache diary covering three consecutive menstrual cycles;
3. Age 18–45 years, with a normal menstrual cycle of 28 ± 7 days and a menstrual duration of 3–7 days;
4. During the 3-cycle screening period, at least 2 of the 3 menstrual cycles have 2–14 days with moderate-to-severe headache (NRS ≥ 4) per cycle (meeting the ICHD-3 appendix criterion for PMM or MRM). Baseline data for the primary outcome will be calculated as the average number of moderate-to-severe headache days across all qualified cycles (i.e., those cycles meeting the 2–14 day criterion) during the screening period;
5. Complete the daily headache diary throughout the 3-cycle screening period (with a completion rate of ≥80% for daily entries within each menstrual cycle) to demonstrate good patient compliance;
6. Voluntary signing of the informed consent form.

#### Exclusion criteria

1. Primary diagnosis of other primary or secondary headache disorders (e.g., tension-type headache, cluster headache, migraine with aura, medication-overuse headache);
2. Concurrent severe or unstable cardiovascular, cerebrovascular, hepatic, renal, or hematopoietic system diseases, or malignant tumors;
3. Diagnosed with severe mental disorders such as major depressive disorder, anxiety disorder, or bipolar disorder;
4. Currently pregnant, breastfeeding, or planning to become pregnant within the next 6 months;
5. Use of hormonal contraceptives or other hormonal therapies affecting the menstrual cycle within the past 3 months; regular use of pain relievers, triptans, or ergotamine-based acute migraine medications for ≥10 days per month within the past 3 months; or receipt of migraine preventive medications, acupuncture, or other systemic non-pharmacological treatments within the past month;
6. Presence of coagulation disorders, severe osteoporosis, or skin infection/lesions at the proposed acupuncture site; history of allergy or severe adverse reactions to acupuncture or press-needle therapy;
7. Currently participating in other interventional clinical trials;
8. Allergy to naproxen sodium or other NSAIDs; history of active gastrointestinal ulcers or gastrointestinal bleeding; or severe hepatic or renal insufficiency;
9. Other conditions deemed by the investigator to preclude participation in the trial or pose significant safety risks.

#### Criteria for discontinuation of intervention

1. Occurrence of a serious adverse event (SAE) related to the study intervention;
2. Confirmed pregnancy during the trial;
3. Significant worsening of headache requiring emergency treatment or alternative therapy, as determined by the investigator;
4. Voluntary withdrawal of consent by the participant;
5. Lost to follow-up, with no contact possible for assessments or interventions;
6. Major protocol violation (e.g., use of prohibited medications, non-adherence to intervention, compromising efficacy or safety evaluation);
7. Development of a new medical condition contraindicating continued trial participation.

#### Management of excluded, withdrawn, and discontinued cases

1. Documentation and Follow-up: All withdrawals or discontinuations will be fully documented. A follow-up will be performed to identify the reason for discontinuation. All previously collected data will be retained for analysis;
2. Data Handling: Participants completing less than 50% of scheduled interventions will be classified as major protocol violators. Their data will be excluded from per-protocol analysis but may be included in intention-to-treat analysis for sensitivity testing;
3. Adverse Event Management: Participants discontinuing due to adverse events or insufficient response will receive timely clinical care or referral as needed;
4. Post-Trial Care: If the study intervention is ineffective, alternative treatment options may be discussed with the participant within ethical guidelines;
5. Sample Size Maintenance: If the dropout rate exceeds the pre-specified threshold, additional participants will be recruited to maintain statistical power.

#### Strategies to ensure participant compliance

1. Informed Consent: Participants will receive a detailed explanation of the protocol, potential risks, adverse events, and management strategies before providing written informed consent;
2. Regular Communication: The research team will maintain consistent contact, address questions promptly, and support participants throughout the trial;
3. Standardized Care: A comfortable treatment environment and consistent, high-quality intervention will be provided. All investigators and operators will complete standardized training;
4. Adherence Monitoring: For the press-needle group, self-pressure and treatment completion will be recorded in diaries. For the naproxen sodium group, medication intake will be documented daily and verified via telephone follow-ups to ensure compliance.

### Interventions

#### Experimental group: sequential press-needle therapy

##### Acupoints and sequential regimen

A phase-specific acupoint regimen will be applied for 3 consecutive menstrual cycles. Each cycle includes 4 treatment sessions in 2 phases:

Fixed acupoint (both phases): Fengchi (GB20), to dispel wind, unblock meridians, and relieve headache.

**Figure 2:**
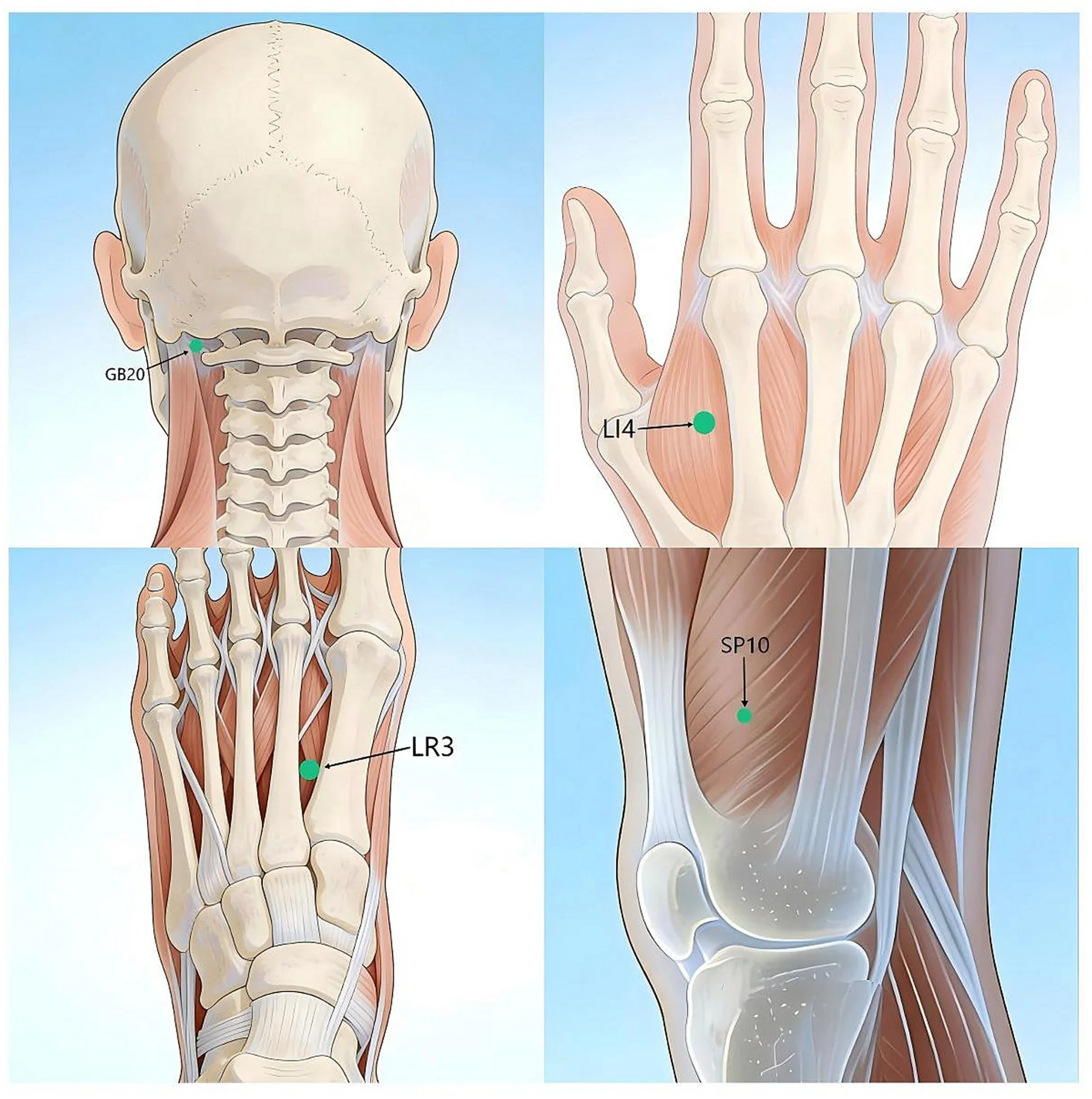
Premenstrual phase acupoint diagram.

Premenstrual phase (2 sessions, 5–7 days before expected menses): GB20 + Taichong (LR3), Hegu (LI4), Xuehai (SP10), to soothe the liver, activate blood circulation, and relieve pain. Acupoint locations are detailed in Table 1 and Figure 2.

**Table 1:** Acupoint location and functional rationale.

Acupoint name	Meridian	Localization (WHO standard)	Traditional rationale
Sanyinjiao (SP 6)	Spleen	On the medial aspect of the leg. 3 cun superior to the tip of the medial malleolus, in a depression close to the medial border of the tibia.	As the confluence point of the Liver, Spleen, and Kidney meridians, it is the primary point for regulating menstruation, nourishing Blood and Yin, and relieving pain in the lower abdomen.
Hegu (LI 4)	Large Intestine	On the dorsum of the hand. Between the 1st and 2nd metacarpal bones, at the midpoint of the radial side of the 2nd metacarpal bone, in the belly of the first dorsal interosseous muscle.	A major source (Yuan) point with potent analgesic effects, especially for disorders of the face and head. It regulates Qi and Blood circulation and disperses pathogenic factors.
Taichong (LR3)	Stomach	On the dorsum of the foot, in a depression distal to the junction of the 1st and 2nd metatarsal bones.	The source (Yuan) and stream (Shu) point of the Liver channel. It pacifies the Liver, subdues Yang, regulates Qi, alleviates depression, and relieves pain. It is crucial for managing conditions related to emotional stress and autonomic dysregulation.
Fengchi (GB20)	Gallbladder Meridian	In the posterior aspect of the neck, below the occipital bone, in the depression between the origins of the sternocleidomastoid and the trapezius muscles.	Its name translates to “Wind Pool.” It is a key point for expelling Wind (a pathogenic factor in TCM associated with sudden onset disorders), clearing the head and eyes, and relieving pain and stiffness in the neck and head. It is a major local point for treating all types of headache.
Zusanli (ST36)	Stomach Meridian	On the anterior aspect of the leg, on the line connecting the depression lateral to the patellar ligament (ST35) with the lateral condyle of the tibia. 3 cun inferior to ST35, one finger-width (middle finger) lateral from the anterior crest of the tibia.	As the He-Sea point and Lower He-Sea point of the Stomach, it is a principal point for regulating the digestive system, strengthening Qi and Blood, and enhancing overall vitality. It is widely used to treat gastrointestinal disorders, fatigue, and to support recovery from chronic illnesses.
Taixi (KI3)	Kidney Meridian	On the medial aspect of the foot, in the depression between the prominence of the medial malleolus and the calcaneal tendon (Achilles tendon), level with the tip of the medial malleolus.	As the Yuan-Source point and Shu-Stream point of the Kidney Meridian, it is the most important point for nourishing Kidney Yin and Yang, replenishing Essence (Jing), and regulating water metabolism. It is indicated for weakness, low back pain, dizziness, tinnitus, and disorders of the reproductive and urinary systems.
Xuehai (SP10)	Spleen Meridian	On the medial aspect of the thigh, 2 cun proximal to the medial superior border of the patella (i.e., 2 cun above the medial patellar angle), on the bulge of the vastus medialis muscle.	Its name means “Sea of Blood.” As a key point on the Spleen Meridian, it regulates Blood circulation, cools Blood, and stops abnormal bleeding. It is the primary point for blood-related disorders such as irregular menstruation, dysmenorrhea, anemia, and skin conditions like eczema and urticaria (hives).

B-cun (Body-cun): The unit of measurement is the individual patient’s own proportional body cun. For limb measurements, the width of the patient’s interphalangeal joint of the thumb is defined as 1 B-cun. Location Verification: Final point location should be confirmed by gentle palpation to identify a tender spot or slight depression before needle application.

**Figure 3:**
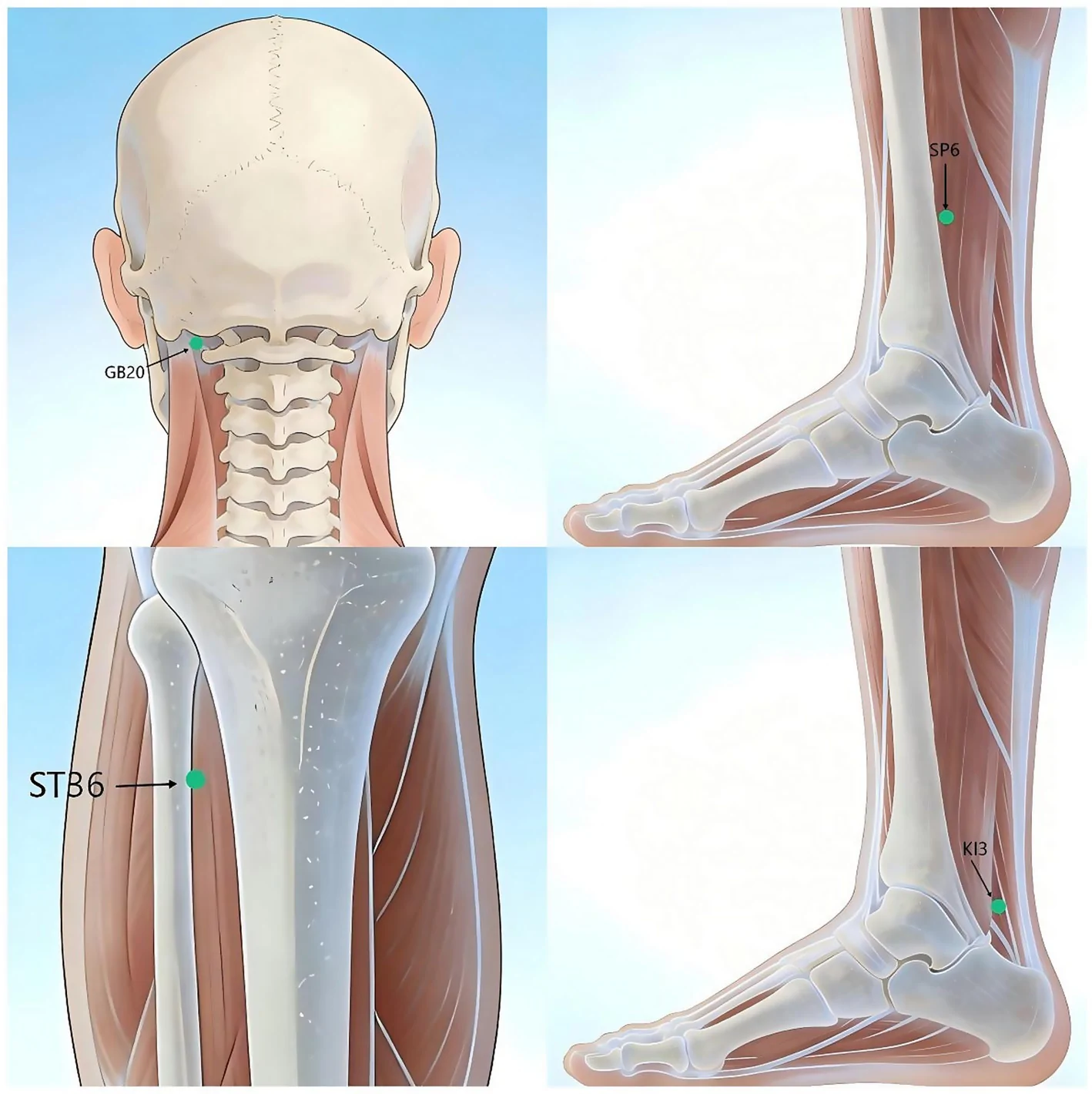
Postmenstrual phase acupoint diagram.

Postmenstrual phase (2 sessions, 2–3 days after menstruation ends): GB20 + Sanyinjiao (SP6), Zusanli (ST36), Taixi (KI3), to nourish liver and kidney, replenish blood, and regulate Chong and Ren meridians. Acupoint locations are detailed in Table 1 and Figure 3.

### Preparation and disinfection

The acupuncturist will wash hands and wear disposable medical gloves. The target skin area will be disinfected with a 70–75% sterile alcohol swab in a centrifugal motion and allowed to air-dry for about 30 s.

### Needle insertion

A disposable sterile press needle (0.18 mm diameter, 1.3 mm length; Suzhou Global Acupuncture Medical Equipment Co., Ltd.) will be used. The needle will be perpendicularly and fully inserted into the skin at the target point with a gentle flicking motion, until the adhesive patch is firmly attached. No additional manipulation is needed.

### Needle retention

The needle will be retained for 48 h. Participants will perform standardized self-pressure and inspect the site daily for redness, swelling, pain, or accidental detachment.

### Removal and disposal

After 48 h, participants will remove the needle and patch before the next visit. Used needles and dressings will be disposed of in designated sharps containers following hospital biohazard protocols.

### Adverse event monitoring

Participants will immediately report persistent local pain, purulence, systemic fever, or complete needle loss. All such events will be documented as adverse events (AEs).

### Standardized self-pressure protocol

Participants will be trained to press the center of the needle pad gently for 1 s per press, 12 repetitions per acupoint per session, 3 sessions daily. Compliance will be monitored via paper diaries, telephone follow-ups, and alarm reminders.

### Control group: oral naproxen sodium therapy

Naproxen sodium was administered for migraine prophylaxis during each menstrual cycle, for 3 consecutive cycles (consistent with the experimental group).

Timing: Initiated 2–3 days before the expected onset of menstruation, and continued until menstruation ended (typically 5–7 consecutive days per cycle).

Dosage: Oral naproxen sodium tablets, 550 mg, twice daily (after breakfast and dinner).

Cycle schedule: Repeated for 3 complete menstrual cycles, aligned with the intervention phases of the press-needle group.

### Compliance monitoring

Medication intake was recorded daily in a medication diary. Adherence was verified via telephone follow-up to ensure correct timing and dosage.

### Quality control

Practitioner Qualifications, Training, and Standardization.

The sequential press-needle intervention will be performed by licensed acupuncturists with at least 10 years of clinical experience.

The naproxen sodium intervention will be dispensed and managed by licensed pharmacists or registered nurses.

All study personnel will complete standardized protocol training before trial initiation, covering intervention procedures, outcome assessment, data recording, and adverse event management. Refresher training will be conducted regularly to ensure consistency.

### Blinding design

This is an open-label trial. Participants and intervention providers will be aware of the group assignments. To avoid detection bias, outcome assessors and statisticians will remain completely unaware of the group assignments throughout the trial. The following measures will be taken to maintain blinding:

(1) Blinded outcome assessors: All outcome assessments will be conducted by trained assessors who were not involved in randomization or treatment administration. Participants will be instructed not to disclose their treatment group to the assessors during any assessment visit;
(2) Blinded statisticians: Randomization codes will not be disclosed to the statisticians until the final data lock-in and completion of all pre-specified analyses. The dataset provided to the statistician will include coded treatment indicators (e.g., Group A and Group B) but will not reveal the correspondence to the actual interventions;
(3) Data manager blinding: Data managers will use coded group identifiers to process and clean the data. They will not have access to randomization information.

To evaluate the influence of the open-label design, participants’ treatment credibility and expectancy will be assessed at baseline and explored as potential confounders in the analysis of treatment effects.

### Treatment fidelity and environmental control

Treatment fidelity will be verified by random audit and standardized record review:

For the press-needle group: documentation of acupoint location, needle retention time, and immediate adverse events.

For the naproxen sodium group: verification of drug dosage, administration timing, and participant adherence. All procedures will be conducted in a quiet, dedicated, and consistent clinical environment to minimize external interference.

### Relevant concomitant care: permitted and prohibited

### Permitted

Rescue medication: Paracetamol (acetaminophen) is allowed as the sole rescue medication for severe migraine attacks. Paracetamol was selected because (1) it has a distinct mechanism of action (central COX-2 inhibition) that does not interfere with the pharmacological effects of the prophylactic NSAID (naproxen sodium, a peripheral COX-1/COX-2 inhibitor); (2) it carries a significantly lower risk of medication-overuse headache (MOH) compared to triptans or combined analgesics (14), which is critical for maintaining the integrity of the primary outcome (headache frequency) over the 3-month intervention period; and (3) it is the most commonly used rescue analgesic in Chinese clinical practice with an established safety profile (15). NSAIDs (including naproxen sodium, except for the assigned prophylactic regimen), triptans, and ergotamines are strictly prohibited to avoid intervention overlap and medication overuse. All rescue medication use (dose and frequency) must be recorded in the daily headache diary, and participants will be instructed to limit paracetamol use to a maximum of 2 days per week to minimize the risk of analgesic overuse.

Non-pharmacological care: Only general lifestyle advice (hydration, sleep, stress management, diet) is permitted; no headache/menstrual-specific non-drug interventions (e.g., acupuncture, moxibustion, herbal medicine) are allowed.

### Prohibited (from screening to final follow-up)

New migraine prophylactic drugs (beta-blockers, anticonvulsants, antidepressants, CGRP monoclonal antibodies) any acupuncture, press-needle, moxibustion, cupping, tuina, or Chinese herbal medicine for headache/menstrual disorders (except the assigned study intervention) new hormonal therapy or procedures altering menstrual cyclicity participation in other interventional clinical trials any other interventions interfering with study outcomes or safety.

### Outcomes

The timeline for evaluating research outcomes at each stage is detailed in Table 2.

**Table 2:** Schedule of outcome assessments across study time points.

Timepoint	Item				
	Baseline	Treatment period			Follow-up period
		1 month	2 months	3 months	3 months post-treatment
Enrollment					
Screening for eligibility	√				
Informed consent	√				
Randomization	√				
Intervention					
Press-needle therapy		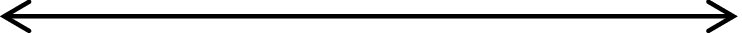			
Sham press-needle therapy		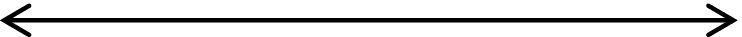			
Assessment					
Primary outcome					
Headache days (e-diary)	√	√	√	√	√
Secondary outcome					
Headache intensity (NRS, e-diary)	√	√	√	√	√
MMFIQ	√	√	√	√	√
MSQ v2.1	√	√	√	√	√
Acute medication use (e-diary)	√	√	√	√	√
HRV recording	√	√	√	√	√
Exploratory outcomes					
Blood biomarkers (CGRP, E2, P4)	√	√	√	√	
Neuroimaging (subset, MRI)	√	√	√	√	
Other measures					
Blinding assessment		√	√	√	√
CEQ	√				
Adverse events monitoring		√	√	√	√
Protocol compliance check		√	√	√	
Concomitant medication review	√	√	√	√	√

NRS, Numeric Rating Scale; MMFIQ, Menstrual Migraine Functional Impact Questionnaire; MSQ, Migraine-Specific Quality of Life Questionnaire; HRV, Heart Rate Variability; CGRP, Calcitonin Gene-Related Peptide; E2, Estradiol; P4, Progesterone. CEQ, Credibility/Expectancy Questionnaire.

### Primary outcome

The primary outcome measure is the change in headache frequency, defined as:

Measurement: The mean change in the number of moderate-to-severe headache days per menstrual cycle from baseline (defined as the average across all qualified cycles during the 3-cycle screening period) to the end of the 3-month intervention period. Definition: A headache day is defined as any calendar day on which the participant experiences a headache meeting ICHD-3 criteria for migraine without aura (ICHD-3 code 1.1) with a peak pain intensity of ≥4 on the 0–10 Numeric Rating Scale (NRS, where 0 = no pain, 1–3 = mild, 4–6 = moderate, and 7–10 = severe; thus an NRS score of ≥4 captures both moderate and severe intensity levels). Data Source: Prospectively captured using a validated, electronic daily headache diary.

### Secondary outcomes

Secondary outcomes will evaluate headache burden, functional impact, acute medication utilization, and autonomic nervous system function.

#### Headache intensity

Measurement: Change in the average peak pain intensity of menstrual migraine attacks per menstrual cycle.

Tool: 11-point Numeric Rating Scale (NRS), where 0 = “no pain” and 10 = “worst pain imaginable.” Participants will rate peak pain intensity for each migraine attack in the ePRO system-integrated daily headache diary.

Assessment Timepoints: Baseline, Treatment Months 1–3, and 3-month post-treatment follow-up.

Analysis: Mean NRS score per menstrual cycle will be calculated, and between-group changes from baseline will be analyzed.

#### Headache-related functional impairment

Measurement: Change in functional disability specifically attributable to menstrual migraine.

Tool: Validated 7-item Menstrual Migraine Functional Impact Questionnaire (MMFIQ), scored 0–100 (higher scores indicate greater functional impairment).

Assessment Timepoints: Baseline, Treatment Months 1–3, and 3-month post-treatment follow-up.

Analysis: Between-group differences in absolute changes in MMFIQ scores from baseline will be compared.

#### Migraine-specific quality of life

Measurement: Change in disease-specific health-related quality of life.

Tool: Chinese version of the Migraine-Specific Quality of Life Questionnaire version 2.1 (MSQ v2.1), comprising three domains: Role Function-Restrictive (RR), Role Function-Preventive (RP), and Emotional Function (EF). Scores are transformed to a 0–100 scale (higher scores indicate better quality of life).

Assessment Timepoints: Baseline, Treatment Months 1–3, and 3-month post-treatment follow-up.

Analysis: Domain-specific and total scores will be computed, with between-group changes from baseline analyzed.

#### Acute medication use

Measurement: Change in the number of days of acute migraine medication use per menstrual cycle.

Tool: Electronic daily headache diary within the ePRO system. Permitted rescue medications are pre-specified.

Assessment Timepoints: Baseline, Treatment Months 1–3, and 3-month post-treatment follow-up.

Analysis: Total number of days of acute medication use per cycle will be summarized, and between-group changes from baseline will be evaluated.

#### Core autonomic function parameters

Measurement: Changes in heart rate variability (HRV) indices, reflecting cardiac autonomic modulation.

Procedure: Standardized 5-min supine resting HRV recording will be performed in a quiet, temperature-controlled room following a 15-min acclimation period.

Equipment: High-fidelity, FDA-cleared single-lead electrocardiogram (ECG) monitor with a sampling rate ≥500 Hz.

Assessment Timepoints: Baseline, Treatment Months 1–3, and 3-month post-treatment follow-up.

Analysis: Raw ECG data will undergo artifact correction and spectral analysis using specialized software. Primary HRV metrics include:

Time-domain: Standard Deviation of NN intervals (SDNN, ms);

Frequency-domain: Low-Frequency power (LF, 0.04–0.15 Hz), High-Frequency power (HF, 0.15–0.40 Hz) in normalized units, and LF/HF ratio.

### Exploratory outcomes

#### Mechanistic biomarkers (blood)

Sample Collection: Peripheral venous blood samples (≈10 mL) will be collected at two phase-specific timepoints per menstrual cycle to account for hormonal fluctuations:

Early follicular phase (menstrual days 2–4);

Mid-luteal phase (≈7 days prior to next expected menstruation, confirmed by cycle tracking or urinary luteinizing hormone kits).

Assessment Timepoints: Baseline and end of Treatment Months 1–3.

Sample Processing and Storage: Blood samples will be centrifuged at 4 °C within 30 min of collection. Serum and plasma aliquots will be stored at −80 °C in a dedicated biobank until batch analysis to minimize inter-assay variability.

Biomarker Analysis: Validated commercial assay kits will be used for quantitative detection:

Calcitonin Gene-Related Peptide (CGRP): High-sensitivity enzyme-linked immunosorbent assay;

Estradiol (E2) and Progesterone (P4): Electrochemiluminescence immunoassay on an automated clinical analyzer.

#### Neuroimaging markers

Procedure: Structural and functional brain MRI will be performed at baseline and end of the 3-month intervention.

Image Acquisition: All scans will be conducted on a 3.0 Tesla MRI scanner using a standardized protocol, including:

High-Resolution 3D T1-Weighted Imaging: For voxel-based morphometry (VBM) analysis to evaluate regional gray matter volume changes in pain-processing (e.g., insula, anterior cingulate cortex) and autonomic regulation (e.g., hypothalamus, brainstem) regions;

Resting-State Functional MRI (rs-fMRI): 8-min eyes-closed scan optimized for blood-oxygen-level-dependent (BOLD) signal detection.

Assessment Timepoints: Baseline and end of Treatment Months 1–3.

Image Analysis: Preprocessing (motion correction, spatial normalization, smoothing) and statistical analysis will follow standard pipelines. Seed-based correlation analysis and independent component analysis (ICA) will evaluate functional connectivity alterations within/between the Salience Network and Central Autonomic Network.

#### Comprehensive mechanism analysis

Correlation analysis using Pearson or Spearman will explore the relationships among the following items: changes in major clinical indicators, such as the number of days with moderate to severe headache; core autonomic nerve indicators, such as the LF/HF ratio; serum biomarker levels, such as calcitonin gene-related peptide, estradiol, P4; and indicators derived from neuroimaging, such as the functional connectivity strength in the target network.

#### Assessment of treatment credibility and expectations

At baseline, validated scales (such as the Credibility/Expectancy Questionnaire) will be used to assess participants’ credibility and expectations regarding their assigned treatment. This assessment will be conducted after randomization and before the first treatment session. The questionnaire consists of two subscales: credibility (how reasonable and helpful the treatment appears) and expectations (the extent of improvement the participant anticipates). These scores will be used in exploratory analyses to examine whether baseline expectations moderate treatment response and will be included as covariates in sensitivity analyses to control for potential bias.

### Safety outcomes

All adverse events (AEs) and serious adverse events (SAEs) were recorded from the time of signed informed consent until the 3-month follow-up after intervention. The occurrence time, symptoms, severity, duration, management, outcome, and causality with interventions were documented in detail.

Sequential press-needle group: Local skin reactions at the press-needle insertion sites were mainly monitored, including redness, itching, pain, rash, infection, and bleeding.

Oral naproxen sodium group: Adverse reactions related to NSAIDs were intensively monitored, including gastrointestinal reactions (nausea, stomachache, acid reflux, abdominal distension, gastrointestinal discomfort), neurological reactions (dizziness, headache, fatigue), skin allergy (rash, itching), and abnormal liver and renal function.

All systemic AEs were recorded in both groups. The incidence, severity, and causality of AEs were compared between groups to comprehensively evaluate the safety of the interventions.

### Sample size

As this is a pilot feasibility study designed to estimate the effect size and variance of the primary outcome for a future definitive trial, no formal hypothesis-testing power calculation was performed. The sample size was determined with reference to previous multicenter randomized controlled trial of acupuncture for menstruation-related migraine (16, 17), which provided reference effect size estimates and variability data for this population. Based on the effect size reported in previous high-quality trials and clinical feasibility, we determined that 30 participants per group would provide a valid estimate of treatment response, variability, and retention (18). Assuming a 20% dropout rate during the trial, we planned to enroll 38 participants per group, with a total target sample size of 76. This sample size is sufficient to estimate key feasibility parameters and provides a preliminary basis for the design and sample size calculation of future confirmatory trials.

### Recruitment

#### Recruitment strategy

Participant recruitment will be conducted at the Chengdu Pidu District Hospital of Traditional Chinese Medicine over an estimated period of 12 months. A multi-pronged strategy will be employed to ensure efficient and ethical enrollment:

Clinical Referral: Primary recruitment will occur through the hospital’s Neurology, Gynecology, and Acupuncture outpatient departments. Board-certified neurologists, gynecologists, and acupuncturists will be informed about the study and will identify potential eligible patients during routine consultations.

Hospital advertising: Research posters and brochures that have undergone ethical review and include key inclusion criteria and contact information will be displayed in prominent locations within the hospital, such as waiting areas and relevant clinical departments.

Community Outreach: Advertisements will be placed on the hospital’s official website and social media platforms (e.g., WeChat public account), targeting women of reproductive age in the local community. All advertisements will be reviewed and approved by the IRB.

#### Recruitment process

The recruitment process will follow a standardized, stepwise procedure to ensure proper screening and informed consent:

Initial Screening: Potentially eligible individuals expressing interest will undergo a preliminary screening, usually via phone or in-person, using a standardized checklist based on the core inclusion/exclusion criteria (e.g., age, headache diagnosis, menstrual cycle regularity).

Informed Consent: Eligible participants will receive a detailed informed consent form approved by the Institutional Review Board. A trained researcher will explain the purpose of the study, the study procedures, potential risks and benefits, the randomization method, and the participant’s right to withdraw from the study at any time without compromising their medical care. Participants will be given ample time (≥24 h) to consider whether to participate and to ask questions before signing the written informed consent form.

Enrollment and Screening Phase: After signing the informed consent form, participants will be formally enrolled and assigned a unique study identification number. Subsequently, they will enter a three-cycle screening phase lasting approximately 12 weeks, during which they must complete a daily headache diary to confirm a diagnosis of PMM or MRM based on the ICHD-3 appendix criteria. During this screening phase, participants will undergo blood draws at specific phases (early follicular and mid-luteal) in each of the qualified cycles, and heart rate variability monitoring.

Diagnostic Confirmation and Baseline Data Extraction: Once participants have completed headache diary entries for all three cycles, two independent neurologists will review the diary data to confirm the diagnosis of PMM or MRM. Baseline data for the primary outcome measures will be extracted from the headache diary of the qualified screening cycles (averaged) only after the diagnosis has been confirmed. Participants who do not meet the diagnostic criteria will be excluded from randomization.

Randomization: Eligible participants who have completed the screening period and whose prospective diary data confirm the diagnosis of PMM or MRM (i.e., at least 2 of the 3 screening cycles meet the predefined headache frequency criterion of 2–14 moderate-to-severe headache days) will be randomly allocated in a 1:1 ratio to the press-needle group or the naproxen sodium group. Participants who do not meet this diagnostic confirmation will be excluded from randomization. Baseline values for the primary outcome will be derived from the average of all qualified screening cycles (those with 2–14 moderate-to-severe headache days), ensuring that the baseline reflects the participant’s typical headache burden rather than a single potentially atypical cycle.

#### Risk–benefit assessment

The risks associated with trial participation are considered minimal to low, as all procedures (press-needle therapy, blood sampling, questionnaires, autonomic and optional imaging assessments) are routine and possess well-established safety profiles. Potential direct benefits for participants include headache relief and improved quality of life, while the study aims to generate valuable scientific knowledge. The favorable risk–benefit profile has been approved by the IRB. All potential risks and benefits are thoroughly disclosed in the informed consent process.

#### Allocation of interventions

Participants who meet all eligibility criteria and provide written informed consent will be formally enrolled and assigned a unique study identification number. A computerized block randomization sequence with a 1:1 allocation ratio (Experimental Group: Control Group) will be generated by an independent statistician not involved in recruitment or intervention delivery, using random block sizes of 4 and 6. The allocation sequence will be concealed using sequentially numbered, opaque, sealed envelopes (SNOSE). Upon enrollment and completion of baseline assessments, the treating acupuncturist will open the next sequentially numbered envelope in the presence of the participant to reveal the group assignment. This process ensures allocation concealment.

#### Data collection and management

Data will be collected using a combination of electronic and paper-based Case Report Forms (eCRFs/pCRFs).

Electronic Data: Primary outcome data (daily headache diary) will be collected via a secure, validated electronic patient-reported outcome (ePRO) system with daily reminders. HRV raw data will be exported from the dedicated device software.

Paper-based Forms: Secondary outcome questionnaires (MMFIQ, MSQ) and safety monitoring forms will be completed on paper during clinic visits and subsequently entered into a dedicated Electronic Data Capture (EDC) system by trained research staff.

Data Management: All data will be entered twice (double data entry) into a password-protected, audit-trail enabled EDC system (e.g., REDCap). Range and logic checks will be programmed to ensure data quality. Regular data monitoring visits will be conducted to verify source data against eCRFs. The final trial database will be locked after resolution of all queries and before statistical analysis.

#### Quality control

Standardized Training: All investigators and research staff will undergo protocol-specific training prior to trial initiation, covering recruitment procedures, intervention protocols, outcome assessment, data entry, and Good Clinical Practice (GCP) guidelines.

Treatment Fidelity: Acupuncturists will follow a detailed treatment manual. Random audits of treatment sessions (e.g., photo verification of point location) will be performed.

Data Monitoring: An independent Data Monitoring Committee (DMC) is not established for this low-risk trial. Instead, the principal investigator and study coordinator will perform regular internal data reviews for completeness, consistency, and protocol adherence.

Protocol Adherence: Regular investigator meetings will be held to discuss protocol adherence and address any operational issues.

#### Confidentiality and ethics compliance

Confidentiality: All participant data will be identified only by a unique study ID. Personal identifying information will be stored separately from study data in a password-protected file accessible only to authorized personnel. All documents will be stored in locked cabinets or on secure servers.

Ethics Compliance: This protocol has been approved by the Ethics Committee of Chengdu Pidu District Hospital of Traditional Chinese Medicine (Approval No. K-2026-016). The trial will be conducted in full accordance with the principles of the Declaration of Helsinki and Chinese regulatory requirements. Any protocol amendments will require prior IRB approval.

#### Statistical methods

All analyses will be performed using both Intention-to-Treat (ITT) and Per-Protocol (PP) principles. A two-sided *p* < 0.05 will be considered statistically significant.

#### Analysis populations

Intention-to-Treat (ITT) Population: All randomized participants, analyzed according to their originally assigned group, regardless of protocol adherence or dropout.

Per-Protocol (PP) Population: A subset of the ITT population comprising participants who completed at least 80% of the planned treatment courses (all three cycles with 4 sessions per cycle), provided valid primary outcome data at the end of the intervention period, and had no major protocol violations.

#### Handling of missing data

The pattern and mechanism of missing data will be examined using descriptive statistics and graphical methods. For the primary outcome, if missing data are judged to be Missing At Random (MAR), multiple imputation (MI) by chained equations will be applied to generate 5 imputed datasets. Auxiliary variables supporting the MAR assumption will include baseline moderate-to-severe headache days, age, migraine subtype (pure menstrual migraine vs. menstrually-related migraine), baseline heart rate variability indices, and treatment adherence levels to improve imputation precision and robustness. Sensitivity analyses will compare MI results with complete-case analysis. To further assess the robustness of results against potential departures from the MAR assumption, a sensitivity analysis under the Missing Not At Random (MNAR) framework will be conducted using a delta-adjusted multiple imputation approach, assuming progressively larger shifts in headache days for participants with missing primary outcome data. For secondary continuous outcomes, mixed-effects models for repeated measures (MMRM) will be used, which provide valid estimates under the MAR assumption using all available data.

#### Handling of multiple comparisons

This trial includes one primary outcome and multiple secondary/exploratory outcomes. The primary outcome is designated as the primary endpoint for this pilot study and will be analyzed at a pre-specified two-sided alpha level of 0.05 without adjustment for multiplicity. Given the exploratory nature of this trial, all results—including the primary outcome—will be interpreted descriptively rather than as confirmatory evidence. No adjustment for multiple comparisons will be performed for any secondary or exploratory outcomes (headache intensity, functional impact, quality of life, acute medication use, heart rate variability indices, serum biomarkers, neuroimaging metrics). All these outcomes are designated as exploratory, and their results will be interpreted strictly as hypothesis-generating rather than confirmatory. This approach prioritizes sensitivity and hypothesis discovery over strict control of the family-wise error rate, and any positive findings will require confirmation in future adequately powered trials.

#### Descriptive statistics

Baseline demographic and clinical characteristics will be summarized by treatment group. Continuous variables will be presented as mean ± standard deviation (SD) or median (interquartile range, IQR) depending on their distribution. Categorical variables will be presented as frequencies and percentages. No formal statistical comparisons of baseline characteristics will be performed, consistent with CONSORT recommendations for randomized trials.

#### Primary outcome analysis

The primary outcome measure—the change in the number of days with moderate-to-severe headaches per menstrual cycle from the last menstrual cycle of the screening period through the end of the 3-month intervention—will be analyzed using a repeated-measure mixed-effects model (MMRM). This model will treat the measurements from each menstrual cycle during the 3-month intervention period as repeated measures, with treatment group, time, and the interaction between treatment and time as fixed effects. The average number of moderate-to-severe headache days from the qualified screening cycles will be included as a covariate, and subject-specific random intercepts will be specified to account for within-subject correlation across the three menstrual cycles.

The primary focus is on the differences in the magnitude of change from baseline across groups at the end of the intervention period (Week 12/Month 3). Results will be presented as adjusted between-group differences, along with their 95% confidence intervals (CI) and corresponding *p*-values. Given that this is a preliminary feasibility study, *p*-values will be interpreted descriptively rather than as confirmatory evidence.

#### Secondary outcomes analysis

Continuous secondary outcomes (e.g., headache intensity assessed by NRS, MMFIQ score, MSQ domains, HRV indices, acute medication use days) will be analyzed using the same MMRM analytical framework as the primary outcome measures and will be adjusted for baseline values. For outcome measures assessed at multiple time points, the same fixed-effects model structure (treatment group, time, and the interaction between treatment and time) will be used.

Given that this trial is a pilot study, all analyses are considered exploratory and are intended to generate hypotheses. Therefore, no correction for multiple comparisons will be performed for any secondary or exploratory outcome measures. All results will be reported with *p*-values, along with effect sizes and 95% confidence intervals. *P*-values will be reported descriptively to indicate the strength of the signal relative to sampling variability.

#### Exploratory and safety analyses

Exploratory Biomarkers/Imaging: Changes in serum biomarker levels (CGRP, estradiol, progesterone) and neuroimaging metrics will be analyzed using paired t-tests or Wilcoxon signed-rank tests within each group, and between-group comparisons will be made using ANCOVA. Correlation analyses (Pearson’s or Spearman’s) will explore relationships between clinical, autonomic, and biomarker/imaging changes. All exploratory findings will be reported with effect sizes and 95% CIs, and will be explicitly labeled as “exploratory, unadjusted for multiple comparisons, for hypothesis generation only” to prevent overinterpretation.

Safety Analyses: The incidence of adverse events (AEs) and serious adverse events (SAEs) will be summarized by treatment group, described by system organ class and preferred term. Differences in AE rates will be compared using Fisher’s exact test.

#### Subgroup and sensitivity analyses

Pre-specified exploratory subgroup analyses will be performed based on menstrual subtype (PMM vs. MRM). Interaction tests between treatment group and menstrual subtype will be performed within the MMRM framework for the primary outcome, with results reported as exploratory (interaction *p*-values reported with 95% CIs). We acknowledge that the trial is not powered for formal subgroup comparisons, and all subgroup findings will be interpreted strictly as hypothesis-generating.

To account for potential expectation effects inherent in the open-label design, baseline “Credibility/Expectation Questionnaire” (CEQ) scores will be collected (19). In the primary analysis, CEQ scores will be included as covariates in the MMRM model to correct for potential expectation-related bias and improve the precision of treatment effect estimates. In addition, exploratory interaction terms (treatment × CEQ score) may be tested to investigate whether baseline expectations moderate treatment response; however, given the pilot nature of this trial, these analyses are strictly hypothesis-generating in nature and will be interpreted descriptively.

Sensitivity analyses will include: (1) PP analysis; (2) ITT analysis without imputation; (3) adjusting for potential baseline imbalances if any are observed.

### Software and reporting

All statistical analyses will be performed using SPSS 29.0 and R 4.4.1. The trial results will be reported in accordance with the Consolidated Standards of Reporting Trials (CONSORT) 2025 statement and its extension for non-pharmacologic treatments and pragmatic trials.

#### Adverse event reporting and harms

All AEs occurring from the time of consent until the end of the study will be recorded, including description, severity, duration, action taken, causality assessment (related/unrelated to intervention), and outcome. SAEs will be reported to the PI and the IRB within 24 h of awareness. The PI will determine causality. An annual safety report will be submitted to the IRB. All AEs will be listed and summarized in the final publication.

#### Plans for collection, laboratory evaluation and storage of biological specimens for genetic or molecular analysis in this trial/future use

Peripheral venous blood samples (10 mL) will be collected at four predefined timepoints early follicular phase (menstrual days 2–4) and mid-luteal phase (approximately 7 days before the next expected menstruation) during the last (third) menstrual cycle of the screening period (serving as the baseline samples), and again at the same two phase-specific timepoints during the final (third) treatment cycle (post-intervention samples). Samples will be processed within 30 min to obtain serum and plasma aliquots, which will be stored at −80 °C in a designated biobank. In the current trial, these specimens will be analyzed for pre-specified biomarkers [calcitonin gene-related peptide (CGRP), estradiol (E2), and progesterone (P4)] using validated commercial assay kits. The baseline biomarker data will be derived from samples collected during the last screening cycle and will be temporally aligned with the baseline headache diary data (derived from the same cycle after diagnosis confirmation). With separate, explicit participant consent, de-identified leftover samples may be retained for future ethically approved research on headache disorders. A secure, coded system will protect participant confidentiality, and participants may withdraw consent for future use at any time.

#### Protocol amendment communication

All protocol amendments must first receive formal approval from the IRB. Once approved, relevant modifications will be promptly updated on the public clinical trial registry. All investigators, study staff, and participants will be formally notified of substantial changes that may affect the study conduct or their welfare.

#### Dissemination of results

The trial results will be submitted for publication in a peer-reviewed scientific journal, regardless of the outcome. Key findings will also be presented at relevant academic conferences and disseminated to the public and patient communities through press releases or institutional media channels to maximize knowledge translation.

## Discussion

The design of our sequential press-needle protocol is grounded in two core rationales: addressing long-term adherence barriers of traditional acupuncture and targeting the phase-specific pathophysiology of menstrual headache via synergistic acupoint selection. First, press-needle therapy was prioritized over conventional manual acupuncture to optimize feasibility—frequent clinic visits, a well-documented barrier to adherence in migraine acupuncture trials (20), are minimized by condensing therapeutic contact to strategically timed needle placement visits. Prolonged stimulation is achieved through indwelling needles with self-administered pressure, integrating treatment into daily life with minimal disruption. This patient-centric design is hypothesized to enhance compliance and satisfaction, critical for managing a chronic cyclical condition like menstrual headache.

The rationale for targeting these phase-specific mechanisms is grounded in the following pathophysiological framework. Menstrual migraine is thought to arise from the complex interplay between ovarian hormone fluctuations and the trigeminovascular system. The most established trigger is the withdrawal of estrogen during the late luteal phase, which is believed to sensitize pain pathways and lower the threshold for migraine initiation. Estrogen modulates multiple molecular cascades relevant to migraine, including serotonergic and glutamatergic neurotransmission, as well as the expression and release of vasoactive neuropeptides (2). Among these, the PACAP pathway has emerged as a critical focus in the pathophysiology of menstrual migraine, with evidence supporting its role in modulating trigeminal and parasympathetic systems, as well as its interaction with hormonal regulation (20). Estrogen fluctuations may also influence the cyclic guanosine monophosphate (cGMP) pathway, which has been implicated in migraine induction through the action of phosphodiesterase inhibitors, and the hormonal milieu may modulate cGMP-mediated nociceptive signaling (21). Similarly, PACAP levels fluctuate across the menstrual cycle, with higher concentrations observed during the perimenstrual and periovulatory phases, which coincide with periods of increased migraine susceptibility (22). These observations raise the possibility that the convergence of estrogen withdrawal with heightened PACAP and cGMP signaling may contribute to the unique clinical features of menstrual migraine—namely, its greater severity, longer duration, and reduced responsiveness to acute treatments compared with non-menstrual attacks. Moreover, the fact that PACAP-induced migraine can occur independently of CGRP receptor activation suggests that multiple parallel pathways may be operative in menstrual migraine, offering potential mechanistic targets for non-pharmacological interventions such as acupuncture (20).

Building upon this mechanistic framework, the acupoint combinations in our protocol were specifically designed for this trial to intervene in these pathways by integrating traditional Chinese medicine theory with modern neuroendocrinology and pain science. The fixed acupoint GB20, located at the base of the skull, can dispel wind and relieve pain (23), acting directly on the trigeminal-cervical nerve complex to alleviate migraines and neck tension (24, 25). To address premenstrual qi stagnation and blood stasis, the acupoints LI4 and LR3 (the “Four Gates”) are selected to soothe the liver, regulate qi, promote blood circulation, and relieve pain (26). Neuroimaging studies have confirmed that these points can modulate pain-related brain networks, improving central sensitization and emotional comorbidities (27, 28); combined with the Xuehai point (SP10) to invigorate blood and resolve stasis (29), modern research shows that SP10 can reduce serum prostaglandin F2α, regulate uterine microcirculation, and alleviate headaches induced by premenstrual vascular dysfunction (30, 31). In the postmenstrual phase, SP6, ST36, and KI3 are used to tonify deficiencies. SP6 is a meeting point of the Liver, Spleen, and Kidney meridians; it regulates menstruation and endocrine function, stabilizing the hypothalamic–pituitary-ovarian axis (32–34); ST36 tonifies qi and blood, enhancing antioxidant and neuroimmune functions (35–37); KI3 nourishes kidney yin, upregulates estrogen receptor expression in the hypothalamus, and restores autonomic nervous system balance (38–40). This protocol combines fixed local acupoints with stage-specific distal acupoints: the premenstrual combination soothes the liver and promotes blood circulation, while the postmenstrual combination tonifies qi, blood, and kidney essence. This approach moves beyond static acupuncture protocols to establish a “time-based acupuncture” model tailored to the circadian rhythms of menstrual headaches.

Mechanistically, we anticipate that this stage-specific intervention will modulate pain-related brain networks and autonomic regulatory circuits. A growing body of neuroimaging evidence suggests that acupuncture can modulate functional connectivity in pain-processing regions, including the amygdala, insula, mesolimbic-parietal cortex, and temporal cortex (41), and can modulate the hypothalamic-limbic-brainstem circuit involved in autonomic control (23, 39, 42). Acupuncture has been shown to enhance connectivity between the amygdala/mediofrontal-parietal cortex and the middle temporal gyrus, as well as between the mediofrontal-parietal cortex and the superior temporal gyrus—regions that play a key role in multisensory pain modulation (41). Furthermore, a meta-analysis of resting-state fMRI studies further confirmed that acupuncture modulates the superior frontal gyrus, angular gyrus, and corpus callosum in migraine patients, and these changes are significantly associated with reduced pain intensity and headache frequency (43). Furthermore, the sustained preventive effects of acupuncture appear to involve influences on the default mode network, the anterior parietal network, the limbic system, and the descending pain modulation system (44). If these imaging changes are observed, they are expected to correlate with reduced headache frequency and improved autonomic balance, providing preliminary evidence for the central mechanisms of sequential pressure acupuncture therapy.

Compared to the control group receiving oral naproxen sodium, the sequential acupuncture therapy in the experimental group—as a non-pharmacological preventive measure—not only demonstrated better tolerability but also allowed for personalized, phased interventions tailored to the cyclical nature of menstrual migraines, aligning closely with the pathophysiological changes of the condition. This therapy avoids the gastrointestinal discomfort, neurological adverse reactions, and potential safety risks associated with long-term use of oral naproxen sodium (45). Unlike existing studies that focus solely on subjective clinical efficacy, this study innovatively integrates multidimensional assessments of objective autonomic nervous system function, serum biomarkers, and neuroimaging to systematically explore the core therapeutic mechanisms of sequential acupuncture therapy.

This trial has several limitations. First, since the preset goal of this study was a feasibility study, the sample size was not determined through formal power calculations for hypothesis testing. Therefore, this trial does not have the statistical power to prove superiority or non-inferiority. All results should be regarded as preliminary and have the significance of generating hypotheses. However, the size of this study can estimate key feasibility parameters and generate effect size estimates, which can provide reference for the sample size calculation of future final clinical trials. Second, the open-label design makes it impossible for participants and intervention providers to be blinded, which may introduce expectancy effects and operational biases. Third, we did not set a sham control group. This decision was based on the fact that the reliable sham operation for 48-h indwelling needles is methodologically challenging, and it may lead to excessive dropout in small sample pre-trials, while the 3-month sham treatment may cause more severe harm to the subjects. Fourth, the simultaneous inclusion of PMM and MRM subtypes introduces clinical heterogeneity, which may affect the interpretation of treatment effects. However, this heterogeneity is intended to enhance the generalizability of the feasibility data in real clinical practice. We have preset stratified subgroup analyses by PMM/MRM subtypes and tested the interaction between the treatment group and the menstrual subtype to explore potential differential responses. All subgroup results are exploratory and need to be verified in larger-scale trials. Fifth, the 3-month intervention period is sufficient for short-term efficacy assessment and evaluation of autonomic nerve function, but it cannot draw conclusions about the long-term sustainability of the treatment effect. Sixth, the single-center design and strict inclusion criteria limit the generalizability of the research results to a wider population, including those with irregular menstrual cycles, users of hormonal contraceptives, and those with prodromal migraine.

Despite certain limitations, this pilot trial demonstrated unique value by integrating multidimensional objective assessments into a specific phase of the press-needle protocol. The feasibility data and projected treatment outcomes from this trial are crucial for the future design of a multicenter confirmatory trial with rigorous statistical power. If the findings of this study are validated, sequential press-needle therapy has the potential to become the evidence-based, non-pharmacological treatment of choice for the prevention and treatment of menstrual migraines, thereby addressing the unmet need in clinical practice for a safe, well-tolerated, and effective treatment strategy.

## Glossary

AE: Adverse Event
ANCOVA: Analysis of Covariance
BOLD: Blood-Oxygen-Level-Dependent
CAN: Central Autonomic Network
CGRP: Calcitonin Gene-Related Peptide
CONSORT: Consolidated Standards of Reporting Trials
CRF: Case Report Form
EDC: Electronic Data Capture
ePRO: Electronic Patient-Reported Outcome
E2: Estradiol
GCP: Good Clinical Practice
HPOA: Hypothalamic–Pituitary-Ovarian Axis
HRV: Heart Rate Variability
ICHD-3: International Classification of Headache Disorders, 3rd edition
IRB: Institutional Review Board
ICA: Independent Component Analysis
ITT: Intention-to-Treat;
LF: Low-Frequency;
HF: High-Frequency;
MH: Menstrual Headache;
MI: Multiple Imputation;
MMFIQ: Menstrual Migraine Functional Impact Questionnaire;
MMRM: Mixed-Effects Models for Repeated Measures;
MRM: Menstrually-Related Migraine;
MSQ: Migraine-Specific Quality of Life Questionnaire;
NRS: Numeric Rating Scale;
NSAID: Non-Steroidal Anti-Inflammatory Drug;
P4: Progesterone;
PMM: Pure Menstrual Migraine;
PP: Per-Protocol;
RCT: Randomized Controlled Trial
rs-fMRI: Resting-State Functional Magnetic Resonance Imaging
SAE: Serious Adverse Event;
SDNN: Standard Deviation of NN Intervals
SN: Salience Network
TCM: Traditional Chinese Medicine
VBM: Voxel-Based Morphometry
